# Folate Receptor: A Macrophage “Achilles' Heel”?

**DOI:** 10.1161/JAHA.112.004036

**Published:** 2012-08-24

**Authors:** Ingo Hilgendorf, Filip K. Swirski

**Affiliations:** Massachusetts General Hospital and Harvard Medical School, Boston, MA

**Keywords:** editorials, atherosclerosis, folate, folate receptor, immunotoxins, macrophages

Cardiovascular diseases are the most common cause of death worldwide.^1^ Atherosclerosis, the underlying inflammatory disease, develops through the progressive accumulation of lipids and leukocytes in the vessel wall. Over the past 30 years, lesional macrophages have emerged as central inflammatory orchestrators of disease progression and its complications. Macrophage precursors, the circulating monocytes, invade predilection sites of dysfunctional endothelium, differentiate, and engulf lipids. Lesional macrophages secrete cytokines, chemokines, growth factors, and lytic enzymes that promote plaque destabilization and rupture, which lead to myocardial infarction and stroke. Interference with monocyte recruitment and macrophage accumulation reduces lesion burden in experimental models, which suggests that such approaches could be beneficial in human disease.^2–4^

Much of current cardiovascular disease management focuses on treatment of known risk factors such as high cholesterol. 3-Hydroxy-3-methylglutaryl coenzyme A reductase inhibitors, the class of drugs known as statins, lower cholesterol but also can exert atheroprotection by targeting inflammation.^5–6^ Treatment of atherothrombotic disease by direct and specific targeting of inflammation is being investigated,^7^ and clinical trials with low-dose methotrexate^8^ and anti–interleukin-1β^9^ are under way. Although these studies are exciting, previous work reminds us that antiinflammatory approaches require caution. Indiscriminate blockade of CD40L showed promise in several experimental studies, but clinical trials had to be aborted because of thromboembolic complications. More selective inhibition of the interaction of CD40L with integrin Mac-1, however, impaired atherogenesis without affecting thrombus formation.^10^ The example illustrates that strategies aimed at targeting inflammation should be considered in their immunophysiological and molecular contexts.

In this issue of the *Journal of the American Heart Association*, Furusho et al^11^ endeavor to treat atherosclerosis by targeting and killing lesional macrophages with an anti–folate receptor β (FRβ) immunotoxin. Macrophages upregulate FRβ during inflammation.^12^ Peritoneal murine macrophages, for example, upregulate FRβ in response to challenge with thioglycollate, zymosan, or bacteria,^13^ and macrophages in rheumatoid arthritis and pulmonary fibrosis express functionally active FRβ that can be targeted therapeutically in an experimental setting.^14–17^ The idea for the study by Furusho et al was therefore simple. If lesional FRβ-expressing macrophages are atherogenic, then delivery of a recombinant immunotoxin (a truncated *Pseudomonas* exotoxin A [PE38] conjugate that kills the infected cell) to those macrophages should be atheroprotective. The anti-FRβ immunotoxin was meant to achieve 3 aims: to target FRβ-expressing cells, to facilitate internalization of the toxin in those cells, and, by implication, to minimize adverse effects of the toxin on bystander, non–FRβ-expressing cells.

Although others have shown in animal models that lesional macrophages upregulate the folate receptor,^18–19^ Furusho et al are the first to target increased FRβ expression on lesional macrophages therapeutically. The authors provide evidence by immunohistochemistry and immunofluorescence that FRβ is expressed by 60% to 70% of CD68-positive lesional macrophages but not by T cells, endothelial cells, or smooth muscle cells. The authors report on FRβ expression in human carotid artery plaques and show that treatment of mice with anti-FRβ immunotoxin decreased lesional macrophage content compared to controls. This reduction in cell number was associated with a reduction in percent lesion area. Importantly, differential blood cell counts did not change in the anti-FRβ immunotoxin group, which suggests a mechanism that is localized and presumably limited to the plaque. Indeed, the authors reported increased cell death in lesions, lending further support that the anti-FRβ immunotoxin acted locally.

Is the study convincing? The effects of the immunotoxin on CD68-positive macrophages are striking, but it is not yet entirely clear how the immunotoxin achieves this. The TUNEL (terminal deoxynucleotidyl transferase dUTP nick end labeling) staining is a clue that the immunotoxin specifically triggers cell death, but no colocalization of TUNEL-positive and FRβ-expressing cells is shown. Moreover, a precise phenotypic analysis of FRβ-expressing cells in atherosclerotic lesions is lacking, and the observation that CD68-positive cells colocalize with FRβ does not preclude the possibility that other important cells are affected.

Despite some uncertainties, the phenotype described in this work is compelling and warrants further work. It remains to be determined whether FRβ-expressing macrophages represent a distinct subset of lesional macrophages or an activation state, as well as whether these macrophages are functionally distinct from their FRβ-negative counterparts. As the authors mention, induction of macrophage apoptosis in atherosclerotic lesions could be a double-edged sword. Although beneficial in early stages of lesion formation, the increased cell death at more advanced stages might be detrimental.^20^ It is also worth considering that folic acid and folate, also known as vitamin B9, are important to many biological functions, such as DNA synthesis and repair. Elimination of cells expressing a particular receptor could have other unforeseen effects that counterbalance the potential benefits observed in this study. Nevertheless, the authors have engineered a clever method to target a formidable cell (Figure 1). The Greek metaphor, though imperfect, can be illustrative: If the macrophage is Achilles and the folate receptor is his Heel, then the antibody is Paris' arrow and the exotoxin the poison that ultimately kills the greatest warrior of the Trojan War.

**Figure 1.**
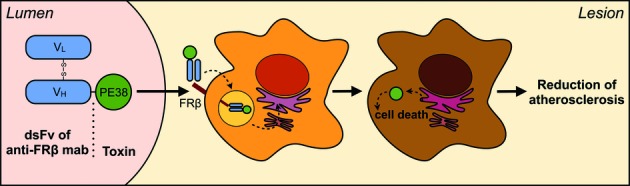
Proposed model of immunotoxin-mediated killing of FRβ-expressing macrophages in atherosclerotic lesions. The immunotoxin consists of a recombinant, biologically active fragment of *Pseudomonas* exotoxin A (PE38) that is linked to the disulfide-stabilized variable region fragment (dsFv) of a monoclonal antibody (mab) against FRβ. Upon binding to FRβ on lesional macrophages, the immunotoxin is internalized and intracellularly processed, leading to toxin-mediated adenosine triphosphate–ribosylation and inactivation of elongation factor 2, inhibition of protein synthesis, and cell death. Elimination of FRβ-positive macrophages reduces atherosclerosis. V_L_ indicates variable region of the light chain; VH, variable region of the heavy chain; and FRβ, anti–folate receptor β.

## References

[1] World Health Organization Global status report of noncommunicable disease. 2010 Available at: http://www.who.int/nmh/publications/ncd_report_full_en.pdf. Accessed July 14, 2012

[2] Stoneman V Braganza D Figg N Mercer J Lang R Goddard M Bennett M Monocyte/macrophage suppression in CD11b diphtheria toxin receptor transgenic mice differentially affects atherogenesis and established plaques. Circ Res. 2007;100:884-893 17322176 10.1161/01.RES.0000260802.75766.00 PMC2040259

[3] Tacke F Alvarez D Kaplan TJ Jakubzick C Spanbroek R Llodra J Garin A Liu J Mack M van Rooijen N Lira SA Habenicht AJ Randolph GJ Monocyte subsets differentially employ CCR2, CCR5, and CX3CR1 to accumulate within atherosclerotic plaques. J Clin Invest. 2007;117:185-194 17200718 10.1172/JCI28549 PMC1716202

[4] Leuschner F Dutta P Gorbatov R Novobrantseva TI Donahoe JS Courties G Lee KM Kim JI Markmann JF Marinelli B Panizzi P Lee WW Iwamoto Y Milstein S Epstein-Barash H Cantley W Wong J Cortez-Retamozo V Newton A Love K Libby P Pittet MJ Swirski FK Koteliansky V Langer R Weissleder R Anderson DG Nahrendorf M Therapeutic siRNA silencing in inflammatory monocytes in mice. Nat Biotechnol. 2011;29:1005-1010 21983520 10.1038/nbt.1989 PMC3212614

[5] Zhou Q Liao JK Pleiotropic effects of statins: basic research and clinical perspectives. Circ J. 2010;74:818-826 20424337 10.1253/circj.cj-10-0110 PMC3807085

[6] Ridker PM Danielson E Fonseca FA Genest J Gotto AMJ Kastelein JJ Koenig W Libby P Lorenzatti AJ MacFadyen JG Nordestgaard BG Shepherd J Willerson JT Glynn RJ Rosuvastatin to prevent vascular events in men and women with elevated C-reactive protein. N Engl J Med. 2008;359:2195-2207 18997196 10.1056/NEJMoa0807646

[7] Charo IF Taub R Anti-inflammatory therapeutics for the treatment of atherosclerosis. Nat Rev Drug Discov. 2011;10:365-376 21532566 10.1038/nrd3444 PMC3947588

[8] Ridker PM Testing the inflammatory hypothesis of atherothrombosis: scientific rationale for the cardiovascular inflammation reduction trial (CIRT). J Thromb Haemost. 2009;7 suppl 1 332-339 19630828 10.1111/j.1538-7836.2009.03404.x

[9] Ridker PM Thuren T Zalewski A Libby P Interleukin-1beta inhibition and the prevention of recurrent cardiovascular events: rationale and design of the Canakinumab Anti-inflammatory Thrombosis Outcomes Study (CANTOS). Am Heart J. 2011;162:597-605 21982649 10.1016/j.ahj.2011.06.012

[10] Wolf D Hohmann JD Wiedemann A Bledzka K Blankenbach H Marchini T Gutte K Zeschky K Bassler N Hoppe N Rodriguez AO Herr N Hilgendorf I Stachon P Willecke F Duerschmied D von zur Muhlen C Soloviev DA Zhang L Bode C Plow EF Libby P Peter K Zirlik A Binding of CD40L to Mac-1's I-domain involves the EQLKKSKTL motif and mediates leukocyte recruitment and atherosclerosis—but does not affect immunity and thrombosis in mice. Circ Res. 2011;109:1269-1279 21998326 10.1161/CIRCRESAHA.111.247684 PMC3291815

[11] Furusho Y Miyata M Matsuyama T Nagai T Li H Akasaki Y Hamada N Miyauchi T Ikeda Y Shirasawa T Ide K Tei C Novel therapy for atherosclerosis using recombinant immunotoxin against folate receptor β–expressing makrophages. J Am Heart Assoc. 2012;1:e003079 doi: 10.1161/JAHA.112.003079 10.1161/JAHA.112.003079 PMC3487340 23130174

[12] Salazar MD Ratnam M The folate receptor: what does it promise in tissue-targeted therapeutics?. Cancer Metastasis Rev. 2007;26:141-152 17333345 10.1007/s10555-007-9048-0

[13] Xia W Hilgenbrink AR Matteson EL Lockwood MB Cheng JX Low PS A functional folate receptor is induced during macrophage activation and can be used to target drugs to activated macrophages. Blood. 2009;113:438-446 18952896 10.1182/blood-2008-04-150789

[14] van der Heijden JW Oerlemans R Dijkmans BA Qi H van der Laken CJ Lems WF Jackman AL Kraan MC Tak PP Ratnam M Jansen G Folate receptor beta as a potential delivery route for novel folate antagonists to macrophages in the synovial tissue of rheumatoid arthritis patients. Arthritis Rheum. 2009;60:12-21 19116913 10.1002/art.24219

[15] Paulos CM Turk MJ Breur GJ Low PS Folate receptor–mediated targeting of therapeutic and imaging agents to activated macrophages in rheumatoid arthritis. Adv Drug Deliv Rev. 2004;56:1205-1217 15094216 10.1016/j.addr.2004.01.012

[16] Nagayoshi R Nagai T Matsushita K Sato K Sunahara N Matsuda T Nakamura T Komiya S Onda M Matsuyama T Effectiveness of anti-folate receptor beta antibody conjugated with truncated *Pseudomonas* exotoxin in the targeting of rheumatoid arthritis synovial macrophages. Arthritis Rheum. 2005;52:2666-2675 16142741 10.1002/art.21228

[17] Nagai T Tanaka M Hasui K Shirahama H Kitajima S Yonezawa S Xu B Matsuyama T Effect of an immunotoxin to folate receptor beta on bleomycin-induced experimental pulmonary fibrosis. Clin Exp Immunol. 2010;161:348-356 20550546 10.1111/j.1365-2249.2010.04182.x PMC2909418

[18] Antohe F Radulescu L Puchianu E Kennedy MD Low PS Simionescu M Increased uptake of folate conjugates by activated macrophages in experimental hyperlipemia. Cell Tissue Res. 2005;320:277-285 15714274 10.1007/s00441-004-1071-7

[19] Ayala-Lopez W Xia W Varghese B Low PS Imaging of atherosclerosis in apoliprotein E knockout mice: targeting of a folate-conjugated radiopharmaceutical to activated macrophages. J Nucl Med. 2010;51:768-774 20395331 10.2967/jnumed.109.071324

[20] Tabas I Macrophage death and defective inflammation resolution in atherosclerosis. Nat Rev Immunol. 2010;10:36-46 19960040 10.1038/nri2675 PMC2854623

